# Health of Louisville

**Published:** 1846-09

**Authors:** 


### HEALTH OF LOUISVILLE.

The Sexton reported the interments in Louisville during the last two weeks in July to have been adults 13, infants 20; and during the firt two weeks of August, adults 12, infants 13; total 57 in the month. Considering that this is in the sickly season, it must be admitted that the report exhibits an excellent state of health in this city. Much sickness has prevailed in certain localities—in all the suburbs, in fact—but it has been sickness of a mild form, chiefly intermittent fever. Cholera infantum has been prevalent, but the slight mortality among children proves that it has not been malignant. A full proportion of the infants died of convulsions, an unusual tendency to which we have remarked in children this season. The convulsions were tetanic in their character. Can there be any connexion between them and the tropical weather, for which the summer just ended has been remarkable? It is not oftener than once in twenty years that such a season as the past is known in our latitude. The heat has been tropical, and the rains have fallen as they do in tropical regions. What will be the consequence of this high temperature and these floods of rain to the health of the city, in the two warm months yet to come, it is impossible to say. In former years, while the city was yet environed by extensive ponds, and large collections of putrid water stood in its streets, the most fatal fevers would have been expected; but Louisville has been now long exempt from such visitations, and, up the present date, it is certain that the extreme heat of the sun and the deluges of rain have not affected the salubrity of our atmosphere.

